# Efficacy of Digital Therapeutics for Pulmonary Rehabilitation: A Multi-Center, Randomized Controlled Trial

**DOI:** 10.3390/life14040469

**Published:** 2024-04-03

**Authors:** Chul Kim, Hee-Eun Choi, Chin Kook Rhee, Jun Hyeong Song, Jae Ha Lee

**Affiliations:** 1 Department of Rehabilitation Medicine, Inje University Sanggye Paik Hospital, Inje University College of Medicine, Seoul 01757, Republic of Korea; josephck@naver.com (C.K.); s5672@paik.ac.kr (J.H.S.); 2 Department of Physical Medicine and Rehabilitation, Haeundae Paik Hospital, Inje University College of Medicine, Busan 48108, Republic of Korea; grace.choi@snsmd.com; 3 Share and Service Inc., Busan 48002, Republic of Korea; 4 Division of Pulmonary and Critical Care Medicine, Department of Internal Medicine, Seoul St. Mary’s Hospital, College of Medicine, The Catholic University of Korea, Seoul 02812, Republic of Korea; chinkook77@gmail.com; 5 Division of Pulmonology and Critical Care Medicine, Department of Internal Medicine, Inje University Haeundae Paik Hospital, Inje University College of Medicine, Busan 48108, Republic of Korea

**Keywords:** rehabilitation, digital health, therapeutics, chronic respiratory disease

## Abstract

Background: This study aimed to investigate the efficacy and safety of digital therapeutics (DTx), EASYBREATH, for pulmonary rehabilitation (PR) in patients with chronic respiratory diseases (CRDs). Materials and Methods: This prospective randomized controlled trial was conducted at multiple centers. Participants were randomly allocated 1:1 to the DTx group (DTxG), provided with DTx using EASYBREATH. The DTxG underwent an 8-week PR program with evaluations conducted at baseline, four weeks, and eight weeks. The control group (CG) underwent one PR session and was advised to exercise and undergo the same evaluation. The primary outcome was the change in six-minute walking distance (6MWD) over eight weeks, and secondary outcomes included changes in scores of Modified Medical Research Council (mMRC), chronic obstructive pulmonary disease assessment test (CAT), and St. George’s respiratory questionnaire (SGRQ). Results: The change in 6MWD after eight weeks demonstrated a significant difference between the DTxG and CG (57.68 m vs. 21.71 m, *p* = 0.0008). The change in mMRC scores (*p* = 0.0008), CAT scores (*p* < 0.0001), and total SGRQ scores (*p* = 0.0003) also showed a significant difference between the groups after eight weeks. Conclusions: EASYBREATH significantly improved exercise capacity, alleviated dyspnea, and enhanced the overall quality of life at eight weeks. EASYBREATH is a highly accessible, time-efficient, and effective treatment option for CRD with high compliance.

## 1. Introduction

Chronic respiratory diseases (CRDs) are among the most common non-communicable diseases globally due to noxious environmental, behavioral, and occupational inhalational exposure [1]. Data from the Global Burden of Diseases, Injuries, and Risk Factors Study (GBD) 2019 showed that approximately 454 million people worldwide were afflicted with CRD in 2019, representing a 39.8% increase compared to 1990 [2]. GBD 2019 also reported that CRDs constituted 4.0 million deaths in 2019 (a 28.5% increase since 1990) and were responsible for 1293.7 disability-adjusted life-years (DALYs) per 100,000 individuals (103 million DALYs among all ages, a 20.8% increase since 1990) [2]. These findings confirm that CRD is common and associated with considerable morbidity and mortality. There is an increasing disease burden on individuals and their caregivers and an economic burden on healthcare systems, which are affected by factors such as the severity of chronic obstructive pulmonary disease (COPD) symptoms (frequent acute exacerbations leading to hospitalization) and the presence of other morbidities, which occur in 30–57% of people with COPD [3]. Therefore, there is a need to address CRD’s growing global burden.

Pulmonary rehabilitation (PR) is an essential treatment for the non-pharmacological management of CRD [4]. It has been proven to reduce dyspnea and improve exercise capacity and quality of life in patients with CRD [5]. Currently, PR is considered an interdisciplinary intervention rather than a multidisciplinary approach for patients with CRD [6]. Furthermore, the 2006 definition highlighted the importance of stabilizing or reversing systemic manifestations of the disease without focusing on behavioral changes.

Based on current insights, the American Thoracic Society (ATS) and the European Union Respiratory Society (ERS) have approved this new definition of PR: “PR is a comprehensive intervention based on patient assessment followed by patient-tailored treatment, which include but are not limited to, exercise training, behavior change and breathing retraining, and, designed to improve the multifaceted condition of people with CRD and promote the long-term adherence of health-enhancing behaviors” [7].

PR improves physical activity and functional performance [4] and greater activity levels are correlated with a reduced risk of hospitalization [8]. Studies have emphasized the importance of maintaining physical activity (PA) in patients with COPD, showing that significantly higher survival rates are associated with increased PA [9,10]. Therefore, it is impossible to improve the aerobic exercise capacity and respiratory muscle strength of patients with CRD using only medications.

Despite its clear benefits, PR is underutilized worldwide and frequently inaccessible to patients. The gap between the advantage of PR and its utilization involves several factors: lack of payers and patients; caregiver awareness and knowledge concerning the necessity, benefits, and process of PR; lack of specialized hospitals providing structured PR and healthcare professionals; insufficient funding; limited resources for PR programs; inadequate allocation of health system reimbursement for PR; suboptimal use of PR by suitable patients [11]; and limited training opportunities for PR professionals [7,12].

Moreover, it is often challenging for patients with COPD who have completed a PR program to maintain and integrate the PR program into their daily lives. Consequently, many patients with COPD fail to adhere to regular exercise training after completing PR and receive insufficient instructions to continue their PR program at home [7]. Therefore, this can be a practical problem with PR, and the efficacy of conventional PR is limited by insufficient participation and high dropout rates [13,14]. As the burden on medical services continues to grow, traditional healthcare models are constantly being challenged, and alternative, cost-effective ways of delivering healthcare to a larger cohort of patients are being explored. Indeed, the key message and objective in the recent ATS/ERS policy statement on the practice and delivery of PR is to expand the provision of PR to suitable patients worldwide [15].

Digital therapeutics (DTx) refers to advanced software medical devices that provide evidence-based therapeutic interventions to prevent, manage, and treat diseases or disorders in patients. Recent studies have compared the impact of digital therapy on PR in patients with COPD and demonstrated comparable outcomes regarding exercise capacity and quality of life as measured using the six-minute walk test (6MWT) [16,17].

The EASYBREATH application (app) is available as a PR program and comprises an aerobic exercise training program, breathing retraining, and an educational program. Individual customized aerobic exercise training programs are implemented in compliance with the exercise prescription created through the 6MWT by the EASYBREATH app. Breathing training and PR education are conducted while watching educational videos in the app. To our knowledge, no current evidence supports using DTx following structured PR programs. We hypothesized that using DTx for PR would improve exercise capacity, respiratory symptoms, and quality of life in patients with CRD.

## 2. Materials and Methods

### 2.1. Participants

This prospective, parallel-group, randomized controlled trial was conducted at multiple centers. The research protocol was approved by the Korean Ministry of Food and Drug Safety (Approval No. 1431) to evaluate the efficacy and safety of a DTx for PR, EASYBREATH. This study was approved by the Institutional Review Board of Catholic University Seoul St. Mary’s Hospital (Approval No. KC23DNDS0031), Inje University Haeundae Paik Hospital (Approval No. 2022-12-013-002), and Inje University Sanggye Paik Hospital (Approval No. 2022-11-022-001). Informed consent was obtained from all participants, and the research was performed in accordance with the Declaration of Helsinki. Despite the nature of a prospective and randomized controlled trial (RCT) in this study, the RCT protocol of this study was conducted without being registered in a clinical trial registry. This was done to ensure protocol security of the device in this study as a domestic regulatory clinical trial.

Based on the data of patients who agreed to participate in this study, we selected eligible participants based on the following inclusion and exclusion criteria. The inclusion criteria were as follows.

(1) Diagnosis with COPD, lung cancer, asthma, bronchiectasis, etc., and requiring PR due to respiratory symptoms such as dyspnea or difficulties in daily life.
(2) Obstructive (ratio of forced expiratory volume in one second to forced vital capacity (FEV1/FVC) < 0.7) or restrictive (predicted FVC < 0.8) abnormalities in pulmonary function tests.
(3) No difficulty using mobile applications on equipment such as smartphones or tablet computers and age ≥ 19 years.

The exclusion criteria were poor cognition, previous participation in PR, unstable cardiovascular disease, walking difficulties, and pregnancy.

Three centers specializing in rehabilitation and respiratory medicine in PR recruited the participants. Participants were randomized in a parallel 1:1 ratio to the DTx group (DTxG), provided with DTx, the EASYBREATH app, or the control group (CG) receiving standard care. Randomization was performed using a random number generator computerized with a block randomization method in SAS version 9.4 (SAS Institute, Inc., Cary, NC, USA).

The DTxG completed a PR program with DTx, EASYBREATH, for eight weeks, with evaluation before and four and eight weeks after PR. The non-PR group underwent one exercise training session and was recommended to exercise and undergo the same evaluation before and after eight weeks.

### 2.2. Intervention (DTx for PR; EASYBREATH)

EASYBREATH (Share and Service Inc., EB-S100, E100020.01, Grade 2, Republic of Korea) is a DTx used for PR. This confirmatory clinical trial aims to confirm the clinical effectiveness and safety of EASYBREATH. The EASYBREATH app consists of the 6MWT, an aerobic exercise training program, breathing retraining, and a PR educational program. The EASYBREATH app was designed by Physical Medicine and Rehabilitation (PM&R) physicians and pulmonologists conducting PR, treating and researching patients with CRD for 10–20 years.

The DTxG had a smartwatch, and the EASYBREATH app was installed on their mobile phones. At the hospital, training for users of DTx for PR using the EASYBREATH app was conducted once. Subsequently, the DTxG performed PR at home using the EASYBREATH app, a total of 24 exercises three times weekly for eight weeks. First, the DTxG opened the EASYBREATH app and performed the 6MWT. Upon completing the 6MWT, an individually customized exercise prescription was created for each patient using the app’s unique exercise prescription algorithm. Subsequently, aerobic exercise training was performed according to individual exercise prescriptions. An exercise report was provided upon the completion of each exercise session. Breathing retraining and PR education were conducted while watching videos.

The medical staff web page monitored DTx participation in PR, EASYBREATH, and exercise data. In addition, patients were contacted by phone if they did not meet the predefined minimal adherence criteria (failure to participate in the exercise three times consecutively).

### 2.3. Primary and Secondary Outcomes

The primary outcome was the change in six-minute walking distance (6MWD) before and after the 8-week PR program. The secondary outcome measures included the Modified Medical Research Council (mMRC) dyspnea scale, COPD assessment test (CAT) to evaluate respiratory symptoms, St. George’s Respiratory Questionnaire (SGRQ) to assess the respiratory quality of life, and Hospital Anxiety and Depression Scale (HADS) to assess anxiety and depression at 4- and 8-week post-treatment compared with pre-treatment. Safety was assessed based on the incidence of adverse device events (ADEs) in each arm on completing the study. We also investigated patient compliance.

### 2.4. Statistical Analysis

Data analyses were performed using SAS version 9.4 (SAS Institute, Inc., Cary, NC, USA). Continuous variables were expressed as mean ± standard deviation or median (interquartile range (IQR)) for baseline characteristics, and an independent t-test or Wilcoxon rank-sum test was used. Categorical variables are expressed as number (%), and the chi-square or Fisher’s exact test was used. The primary and secondary outcomes present each group’s descriptive statistics. The significance level was set at *p* < 0.05.

## 3. Results

### 3.1. Demographics and Baseline Parameter

We prospectively enrolled 99 eligible patients between January 2023 and November 2023. Initially, ninety-two patients who met the criteria were enrolled in this study; however, eight patients either withdrew their consent or were lost to follow-up. As a result, 84 patients completed the study, 43 in the DTxG and 41 in the CG.

The mean age of the patients was 65.54 ± 9.07 years. There were no intergroup differences in baseline clinical characteristics except mean age (Table 1). The participants had moderate airflow obstruction, with a mean FEV1 predicted at 63.1% and 70% per group. There were no intergroup differences in the incidence or severity of acute exacerbations. The comorbidities in each intervention arm are shown in Table 2.

### 3.2. Primary Outcome

**Figure 1:**
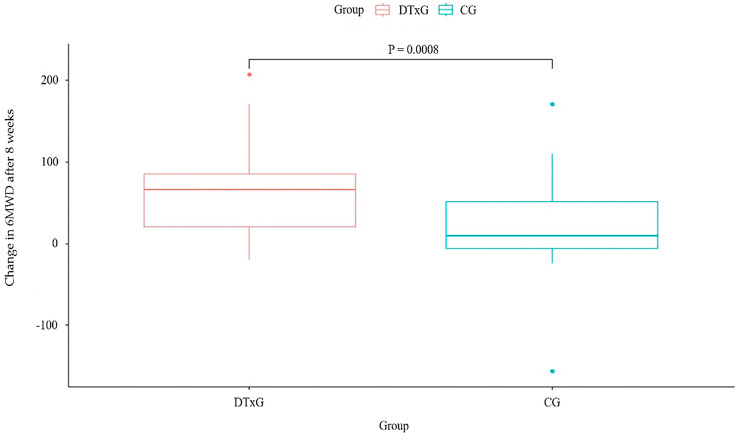
Changes in 6MWD after eight weeks between two groups. 6MWD, six-minute walking distance; DTxG, digital therapeutics group; CG, control group.

Changes in 6MWD over eight weeks of treatment had a significant difference between the DTxG (57.68 ± 56.25) and the CG (21.71 ± 49.70) (*p* = 0.0008) (Figure 1). The mean baseline 6MWD was 495.67 ± 64.15 m in the DTxG and 474.54 ± 73.36 m in the CG, and increased after 8 weeks to 553.35 ± 86.41 m and 496.25 ± 84.07 m, respectively (Table 3). Only the DTxG showed a change of >54 m, the minimum clinically important difference (MCID) [18] of the 6MWD after eight weeks of treatment.

### 3.3. Secondary Outcomes

#### 3.3.1. Modified Medical Research Council

The mMRC showed no intergroup differences at baseline (*p* = 0.8859). However, the change in mMRC had a significant intergroup difference after four and eight weeks of treatment (57.68 ± 56.25 vs. 21.71 ± 49.70, *p* = 0.0008) (Table 4).

#### 3.3.2. COPD Assessment Test

**Figure 2:**
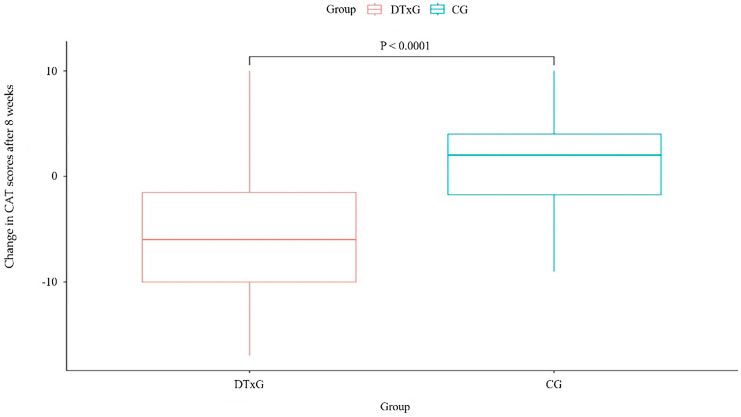
Changes in CAT scores after eight weeks between two groups. CAT, chronic obstructive pulmonary disease assessment test; DTxG, digital therapeutics group; CG, control group.

The change in CAT score after eight weeks of treatment showed a significant intergroup difference (*p* < 0.0001). In the DTxG, CAT scores decreased after eight weeks compared to baseline (mean difference −4.86 ± 7.00); on the other hand, in the CG, CAT scores increased (mean difference 1.29 ± 4.45) (Figure 2 and Table 5). Only the DTxG showed a decrease of >two points, which is the MCID [19] in the CAT score after eight weeks of treatment. Moreover, the change in CAT scores after four weeks of treatment showed a significant intergroup difference (*p* < 0.0001).

#### 3.3.3. St. George’s Respiratory Questionnaire

**Figure 3:**
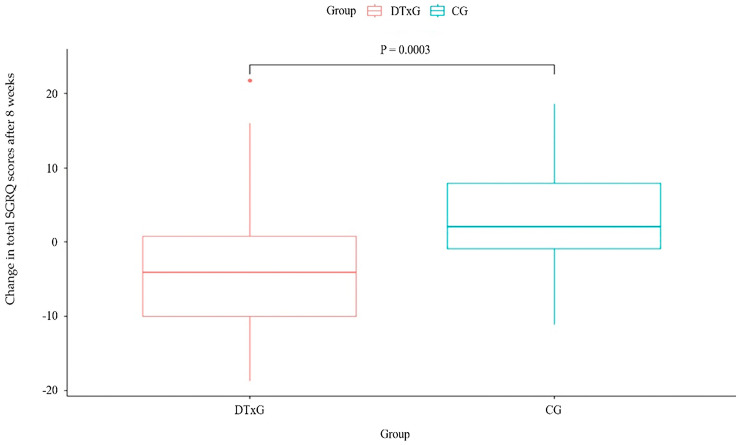
Changes in SGRQ after eight weeks between two groups. SGRQ, St. George’s respiratory questionnaire; DTxG, digital therapeutics group; CG, control group.

The change in the total SGRQ score after eight weeks of treatment showed a significant intergroup difference (*p* = 0.0003) (Figure 3 and Table 5). The SGRQ total score decreased from baseline to eight weeks in the DTxG (mean difference −3.32 ± 9.12) but increased in the CG (mean difference 3.11 ± 6.45). Moreover, the total SGRQ score change after 4 weeks of treatment showed a significant intergroup difference (*p* = 0.0035).

#### 3.3.4. Hospital Anxiety and Depression Scale

The change in the total HADS score after 4 weeks of treatment showed a significant intergroup difference (*p* = 0.0195); the group differences in the changes in total HADS scores were insignificant at eight weeks (Table 5). The total HADS score decreased from baseline to four weeks in the DTxG (mean difference −1.98 ± 4.60) but increased in the CG (mean difference 0.37 ± 4.40).

#### 3.3.5. Safety

Overall, no significant intergroup differences were observed in adverse events. Most of the seventeen cases were not associated with this clinical trial, and two cases in the DTxG were ADEs due to an ankle sprain and fever.

#### 3.3.6. DTx Usage and Compliance

Compliance was evaluated as the percentage of participation in the 24 PR sessions over 2 months, and compliance in the DTxG was very high. Notably, subjects in the DTxG participated in >80% of the 24 sessions. The DTX app usage is defined as follows:

- Very high: ≥80%;
- High: >60% but <80%;
- Normal: >40%~<60%;
- Discomfort: >20%~<40%;
- Very uncomfortable: <20%.

## 4. Discussion

The current study found that the DTxG with EASYBREATH showed significantly better improvements in 6MWD, dyspnea symptoms, and quality of life after eight weeks of intervention than in the CG. The 6MWD and CAT scores after eight weeks of EASYBREATH usage showed changes greater than the MCID. To our knowledge, this is the first multi-center randomized clinical trial to evaluate the efficacy of DTx for PR following an aerobic exercise-based structured PR program to improve aerobic exercise capacity, dyspnea symptoms, and quality of life.

Digital interventions associated with PR for CRD management have been based on behavioral change therapy (BCT) [20]. Evidence from Cochrane reviews does not clearly demonstrate the benefit or harm of digital interventions that support self-management or multi-component interventions in which self-management is a digital component of the intervention for impact on PA, health behaviors, or self-efficacy [20]. These findings, along with those of other reviews, highlight the need for more research to understand the mechanisms underlying the effectiveness of these interventions [20]. Moreover, PR emphasizes the importance of stabilizing or reversing systemic manifestations of the disease without paying specific attention to behavioral changes [6]. Therefore, applying aerobic exercise training, the main action principle of PR, to digital interventions is more crucial than applying BCT.

Exercise training, considered the cornerstone of PR, is the best way to improve exercise capacity and muscle function in CRD [7,21]. Improvements in skeletal muscle function after exercise training increase exercise capacity despite the absence of changes in lung function [22,23]. Aerobic exercise capacity is a vital predictor of mortality in healthy individuals and those with several chronic diseases [9,24]. Exercise training aims to condition the ambulatory muscles and improve aerobic exercise capacity, increasing PA associated with reduced breathlessness and fatigue. High-intensity endurance exercise training is commonly used in PR programs [25]. The Frequency, Intensity, Time, and Type (FITT) framework was recommended by the American College of Sports Medicine (ACSM) Guidelines for Exercise Testing and Prescription, and it can be applied to PR [26]. The general principles of exercise training in patients with CRD do not differ from those of healthy individuals or athletes. For physical training to be effective, the total training load should reflect the specific requirements of the individual exceeding the daily loads to improve aerobic capacity and muscle strength. It must progress as improvement occurs [7]. EASYBREATH implemented a well-structured, individualized PR program based on aerobic exercises. When the 6MWT is performed with EASYBREATH, individual exercise prescriptions are issued through the unique exercise prescription algorithm built into EASYBREATH. Therefore, the subjects exercised while being monitored at an optimal exercise intensity that could effectively and safely improve their aerobic exercise capacity. Therefore, using EASYBREATH for eight weeks resulted in significant changes in 6MWD.

Moreover, the 6MWD was improved to 62.87 m, which was higher than the MCID of 54 m. This was consistent with the results of a Cochrane meta-analysis [4]. The meta-analysis showed that the mean treatment effect of 6MWD was greater than the threshold for clinical significance. This proved that using DTx for PR is superior to recommending self-exercise after PR education without any tools to improve exercise capacity.

Another digital intervention for PR, the Kaia COPD application, comprises an exercise training program, breathing exercises, and an educational program. However, the exercise training program was conducted mainly as a strength training program [17]. The main analysis of this study showed a significant intergroup difference in the change in median steps after six months. However, it does not provide the FITT framework for aerobic exercise recommended by the ACSM, and the number of steps taken does not directly indicate exercise capacity. Fastenau et al. reported an inconsistency between functional exercise capacity and daily PA in patients with mild to moderate COPD enrolled and evaluated in primary care [27].

The online PR program ‘my-PR’ was designed to allow patients to keep up with the video-facilitated exercises in real time. They conducted ten exercises: bicep curls, push-ups against a wall, squats, leg extensions in a sitting position, upright rows with weights, arm punches with weights, sit-to-stand, arm swings with a stick, leg kicks to the side, and step-ups. They reported that a 6-week program of online-supported PR was non-inferior to a conventional face-to-face session regarding effects on 6MWD and symptom scores. Moreover, it was safe and well-tolerated [16]. However, the online program did not provide the FITT framework for aerobic exercises recommended by the ACSM. Video-facilitated exercises were not superior to face-to-face PR, which comprised two supervised sessions for six weeks. Video-facilitated exercises cannot induce individualized exercise, and intensity must be adjusted independently. This seems to limit significant improvements in exercise capacity.

As reported in many PR guidelines and meta-analyses, conventional PR improves exercise capacity, dyspnea, and quality of life [28,29]. As an extension of these research results, DTx for PR, EASYBREATH, also improved exercise capacity, dyspnea, and quality of life after eight weeks. The mMRC showed a significant intergroup difference after eight weeks of treatment. The change in CAT scores after four and eight weeks of treatment showed a significant intergroup difference (*p* < 0.001). CAT scores decreased from baseline to eight weeks in the DTxG but increased in the CG. Only the DTxG decreased by >two points, which was the MCID [19] of the CAT score, after eight weeks of treatment. The change in the total SGRQ score after eight weeks of treatment showed a significant intergroup difference (*p* = 0.0003). The total SGRQ score decreased from baseline to eight weeks in the DTxG but increased in the CG. Moreover, the total SGRQ score change after four weeks of treatment showed a significant intergroup difference (*p* = 0.0035). This finding is consistent with that of a meta-analysis of 19 studies [4]. The meta-analysis also observed significant improvements in total SGRQ scores.

Despite the benefits of health promotion, a significant proportion of eligible patients do not complete rehabilitation programs. In studies that conducted PR for more than seven weeks with a sample size of over 100, incompletion rates were reported to vary from 20% to 40% [30]; however, non-completion rates of >70% have also been reported [31]. Attendance rates during rehabilitation have seldom been reported but appear to vary at approximately 90% for intensive short-term programs (<12 weeks) with three training sessions weekly [32,33]. Regarding the compliance of PR’s digital intervention, 75% of the app’s activity in at least 50% of the trial weeks was 79%, and 61% of users participated in 50% of the trial weeks [17]. Concerning another study, 72% of the two face-to-face PR sessions were attended, compared with 62% of the suggested five sessions recorded as online accessed PR over the 6-week intervention period. In this study, all subjects in the DTxG participated in >80% of the 24 sessions, and the EASYBREATH compliance was high. Based on the above studies, the compliance rate of DTx for PR is not inferior to that of face-to-face PR.

PR is currently one of the most cost-effective treatment strategies [15]. Recent studies reported a long-term reduction in medical costs by reducing the use of healthcare after PR [34,35,36,37]. This means that PR reduces the number or length of hospitalizations and the frequency of acute exacerbations, thereby reducing the number of visits to the emergency room or outpatient clinic [38,39,40,41]. Griffith et al. conducted a randomized controlled study for six weeks and compared patients who received PR for six weeks and those who received general treatment. There was a clear reduction in medical costs through the decrease in healthcare use by analyzing medical costs for 1 year, considering the cost of PR [38]. In addition, a systematic review of the literature showed that patients who received PR after acute exacerbation had decreased readmission rates and number of healthcare uses [42]. Notably, randomized clinical trials have shown that PR is cost-effective [43]. Improving the constraints of PR treatment and taking advantage of the high accessibility and time-efficient benefits of DTx will reduce national medical costs by providing appropriate PR treatment to more patients with CRD.

There are some limitations in our study. First, although this study was a multi-center study, the number of included patients was small, limiting the generalizability of the research results [44,45,46]. Nevertheless, the baseline characteristics and outcomes observed in our study closely paralleled those documented in previous studies. Second, double-blinding was unattainable due to the utilization of smartwatches and apps in the DTx. group, which may render the possibility of performance bias. Third, several pulmonary diseases were included in this study. However, there was no statistically significant difference in the number of subjects for each pulmonary disease in the DTxG and CG. Future studies should consider focusing on COPD patients to minimize disease-related biases. Fourth, age is significantly higher in the CG than in the DTxG. Therefore, an Analysis of Covariance (ANCOVA) was performed to determine whether the age factor affected the difference in the change in walking distance for 6 min after 8 weeks between the DTxG and the CG. As a result of the analysis, the group factor was statistically significant in the change in 6 min walking distance after 8 weeks (F = 11.671, *p* = 0.001), while age was not statistically significant (F = 0.004, *p* = 0.951). Fifth, the PR duration was limited to just eight weeks, posing challenges in confirming its long-term efficacy. Consequently, future studies should investigate the long-term efficacy and adherence of PR utilizing DTx, incorporating an extended treatment duration.

## 5. Conclusions

DTx for PR, EASYBREATH, significantly improved exercise capacity, alleviated dyspnea, and enhanced the overall quality of life at eight weeks. EASYBREATH, a DTx for PR, is a highly accessible, time-efficient, and effective treatment option for CRD with high compliance.

## Figures and Tables

**Table 1:** Participants’ demographics and baseline parameters.

Variables	Total (*n* = 84)	DTxG (*n* = 43)	CG (*n* = 41)	*p*-Value
Age, years	65.54 ± 9.07	63.40 ± 10.36	67.78 ± 6.93	0.0355 ^¥^
Male	70 (83.33)	34 (79.07)	36 (87.80)	0.2829 ^†^
Height, cm	166.48 ± 6.86	167.32 ± 6.93	165.61 ± 6.77	0.2578 *
Body weight, kg	64.88 ± 13.22	64.41 ± 12.14	65.37 ± 14.40	0.7418 *
Resting HR, beats/min	86.14 ± 12.43	86.14 ± 12.43	86.44 ± 9.20	0.9008 *
Resting SBP, mmHg	126.62 ± 15.36	129.40 ± 15.41	123.71 ± 14.94	0.0899 *
Resting DBP, mmHg	76.64 ± 11.04	77.58 ± 10.67	75.66 ± 11.46	0.4282 *
Resting Saturation, %	96.77 ± 1.94	96.72 ± 1.98	96.83 ± 1.92	0.5283 ^¥^
FEV1, % predicted	66.49 ± 17.12	63.10 ± 16.74	69.97 ± 17.02	0.082 ^¥^
FEV1/FVC, %	61.73 ± 15.38	59.28 ± 15.35	64.24 ± 15.21	0.083 ^¥^
Ever smoker	63 (75)	33 (76.74)	30 (73.17)	0.191 ^†^
History of Acute Exacerbation	15 (17.86)	7 (16.28)	8 (19.51)	0.731 ^†^
Incidence of Acute Exacerbation				
Moderate Exacerbation	4 (4.76)	2 (4.65)	2 (4.88)	1.000 ^‡^
Severe Exacerbation	13 (15.48)	5 (11.63)	8 (19.51)	0.097 ^‡^

^†^: Pearson’s chi-square test, ^‡^: Fisher’s exact test, *: Two-sample *t*-test, ^¥^: Wilcoxon’s rank sum test. Data are presented as mean ± SD or number (%) unless otherwise indicated. DTxG, digital therapeutics group; CG, control group; HR, heart rate; SBP, systolic blood pressure; DBP, diastolic blood pressure; FEV1, forced expiratory volume in one second; FVC, forced vital capacity; SD, standard deviation.

**Table 2:** Comorbidities of the participants.

Variables	Total (*n* = 84)	DTxG (*n* = 43)	CG (*n* = 41)
Diabetes mellitus	17	8	9
Hypertension	18	9	9
Liver disease	4	3	1
Tuberculosis	8	3	5
NTM-PD	1	1	0
COPD	49	23	26
GERD	7	2	5
CVA	4	2	2
Heart disease	15	8	7
Kidney disease	3	2	1
Cancer	4	1	3

Data are presented with number. DTxG, digital therapeutics group; CG, control group; NTM-PD, nontuberculous mycobacteria pulmonary disease; COPD, chronic obstructive pulmonary disease; GERD, gastroesophageal reflux disease; CVA, cerebrovascular accident.

**Table 3:** Changes in 6MWD over eight weeks.

6MWD, m	Total (*n* = 84)	DTxG (*n* = 43)	CG (*n* = 41)	*p*-Value
Visit 1 (Pre-treatment)	485.36 ± 69.20	495.67 ± 64.15	474.54 ± 73.36	0.1632 *
Visit 4 (Week 8)	525.48 ± 89.50	553.35 ± 86.41	496.25 ± 84.07	0.0034 ^¥^
Change at Visit 4 (after 8 weeks)	40.12 ± 55.84	57.68 ± 56.25	21.71 ± 49.70	0.0008 ^¥^

*: Two-sample *t*-test; ^¥^: Wilcoxon rank sum test. Data are presented with mean ± SD. 6MWD, six-minute walking distance; DTxG, digital therapeutics group; CG, control group; SD, standard deviation.

**Table 4:** Changes in mMRC scores over eight weeks.

mMRC	Total (*n* = 84)	DTxG (*n* = 43)	CG (*n* = 41)	*p*-Value
Visit 1 (Pre-treatment)	1.31 ± 0.69	1.33 ± 0.71	1.29 ± 0.68	0.8859 *
Visit 3 (Week 4)	1.31 ± 0.64	1.21 ± 0.64	1.41 ± 0.63	0.2099 *
Change at Visit 3 (after 4 weeks)	0.00 ± 0.54	−0.12 ± 0.63	0.12 ± 0.40	0.0212 *
Visit 4 (Week 8)	1.19 ± 0.67	1.00 ± 0.65	1.39 ± 0.63	0.0072 *
Change at Visit 4 (after 8 weeks)	−0.12 ± 0.63	−0.33 ± 0.71	0.10 ± 0.44	0.0008 *

*: Wilcoxon rank sum test. Data are presented with mean ± SD. mMRC, modified medical research council; DTxG, digital therapeutics group; CG, control group; SD, standard deviation.

**Table 5:** Changes in QOL after eight weeks.

	Total (*n* = 84)	DTxG (*n* = 43)	CG (*n* = 41)	*p*-Value
CAT				
Visit 1 (Pre-treatment)	17.65 ± 6.69	17.67 ± 6.03	17.63 ± 7.39	0.9782 *
Visit 3 (Week 4)	16.10 ± 6.97	13.37 ± 5.29	18.95 ± 7.43	0.0002 *
Change at Visit 3 (after 4 weeks)	−1.56 ± 5.33	−4.30 ± 4.79	1.32 ± 4.27	<0.0001 ^¥^
Visit 4 (Week 8)	15.80 ± 7.80	12.81 ± 6.35	18.93 ± 8.02	0.0002 *
Change at Visit 4 (after 8 weeks)	−1.86 ± 6.62	−4.86 ± 7.00	1.29 ± 4.45	<0.0001 ^¥^
SGRQ				
Visit 1 (Pre-treatment)	29.28 ± 13.38	30.06 ± 12.90	28.45 ± 13.99	0.3734 ^¥^
Visit 3 (Week 4)	29.03 ± 13.80	28.06 ± 13.68	30.05 ± 14.02	0.4871 ^¥^
Change at Visit 3 (after 4 weeks)	−0.24 ± 6.58	−2.00 ± 7.69	1.59 ± 4.58	0.0035 ^¥^
Visit 4 (Week 8)	29.09 ± 14.26	26.74 ± 14.51	31.56 ± 13.73	0.0688 ^¥^
Change at Visit 4 (after 8 weeks)	−0.18 ± 8.52	−3.32 ± 9.12	3.11 ± 6.45	0.0003 *
HADS				
Visit 1 (Pre-treatment)	9.54 ± 5.71	9.12 ± 5.62	9.98 ± 5.83	0.4938 *
Visit 3 (Week 4)	8.70 ± 5.68	7.14 ± 5.42	10.34 ± 5.53	0.0093 ^¥^
Change at Visit 3 (after 4 weeks)	−0.83 ± 4.63	−1.98 ± 4.60	0.37 ± 4.40	0.0195 *
Visit 4 (Week 8)	8.35 ± 6.11	7.53 ± 6.03	9.20 ± 6.14	0.2278 ^¥^
Change at Visit 4 (after 8 weeks)	−1.19 ± 4.25	−1.58 ± 4.52	−0.78 ± 3.97	0.3915 *

*: Two-sample *t*-test, ^¥^: Wilcoxon rank sum test. Data are presented as mean ± SD. QOL, quality of life; DTxG, digital therapeutics group; CG, control group; CAT, chronic obstructive pulmonary disease assessment test; SGRQ, St. George’s. respiratory questionnaire; HADS, hospital anxiety and depression scale; SD, standard deviation.

## Data Availability

All the data are contained within the manuscript.
